# Laboratory based surveillance of travel-related *Shigella sonnei *and *Shigella flexneri *in Alberta from 2002 to 2007

**DOI:** 10.1186/1744-8603-6-20

**Published:** 2010-11-01

**Authors:** Steven J Drews, Chris Lau, Marnie Andersen, Christina Ferrato, Kim Simmonds, Liala Stafford, Bev Fisher, Doug Everett, Marie Louie

**Affiliations:** 1 Provincial Laboratory for Public Health (Microbiology)(ProvLab), Calgary, Alberta, Canada; 2 Department of Microbiology and Infectious Diseases, University of Calgary, Calgary, Alberta, Canada; 3 Alberta Health and Wellness, Edmonton, Alberta, Canada

## Abstract

Between 2002 and 2007, travel related cases of *Shigella sonnei *and *S*. *flexneri *in Alberta, Canada were acquired from Central America, the Indian subcontinent and North America. Of this group, resistance to ciprofloxacin and nalidixic acid was identified in isolates from patients who had travelled to the Indian subcontinent. This study provides a Canadian perspective to a growing body of literature linking ciprofloxacin and nalidixic acid resistance to travel to the Indian subcontinent.

*Shigella *is a common cause of diarrheal illness in North America with a rate of 2.0 per 100,000 in Canada [1] and a rate of 3.2 per 100,000 in the United States [2,3]. Imported cases of *Shigella *infections have been reported in developed countries following travel to a foreign or developing country [4,5] and may be impacted by factors including socio-economic factors [6], food distribution networks [5] and microbiologic factors [7]. Across multiple geographic regions, high rates of antimicrobial resistance to multiple agents (e.g. sulfonamides, tetracycline, chloramphenicol, ampicillin, and trimethoprim-sulfamethoxazole) have limited the choices for empiric antimicrobial therapy required to manage *Shigella *infections and reduce fecal excretion of the bacteria [8-10] with descriptions of shifting species dominance and changes in antimicrobial susceptibility [10,11]. Generally, *Shigella flexneri *and *Shigella sonnei *are the dominant species and are heavily impacted by changes in antimicrobial susceptibility [12,13].

This study identifies the global regions associated with travel-related cases of *S*. *flexneri *and *S*. *sonnei *in Alberta, Canada and compares antibiotic resistance patterns of these isolates for 2002 to 2007 inclusive.

Specimens collected 2002-2007 (inclusive) from *S. flexneri *and *S. sonnei *infections in Alberta, Canada were included for study. Data collected at time of specimen submission included: date of specimen collection, outbreak association if present, travel history and antibiogram (data source-ProvLab Information Systems; Communicable Disease Report at Alberta Health and Wellness). Outbreaks were defined by public health officials as ≥ 2 epidemiologically related cases. Each outbreak was assigned a unique incident number. Repeat isolates received within six months of original case infections were excluded. Only one representative case for each outbreak was included, unless the isolates had different antibiotic susceptibility patterns. Based on travel history the origin of an isolate was grouped into corresponding regions and continents. Regions included in the study represented major travel destinations for individuals living in Canada. Domestic exposures were defined as "travel within North America."

## Isolate confirmation

Presumptive *Shigella *isolates were confirmed using conventional biochemical tests [14]. Serotyping was done for *S. flexneri *and phagetyping was done for *S*. *sonnei*. Serotyping was performed using commercially available antisera (Denka Seiken USA Inc., Campbell, CA) for *S .flexneri *and the following serotypes (STs) were determined: 1-4, 6, SH-101, SH-104, and variants × or y [14]. Phage typing was performed on *S. sonnei *isolates following standard procedures at the National Microbiology Laboratory in Winnipeg, Manitoba [15]. For 2002 and 2003, there were representative but fewer numbers of isolates were available for testing. For example, in 2002 and 2003, only 24% and 58% of representative isolates were available respectively. From 2004-2007, representative isolates for each case of infection were available for susceptibility testing: 2004 (100%), 2005 (100%), 2006 (89%), 2007 (91%).

## Susceptibility testing

Susceptibility testing was performed using Sensititre panels (Trek Diagnostic Systems, Cleveland, OH) against the following antimicrobial agents:

• amikacin (AMI)

• amoxicillin/clavulanic acid (AMC)

• ampicillin (AMP)

• cefoxitin (FOX)

• ceftiofur (TIO)

• ceftriaxone (AXO)

• chloramphenicol (CHL)

• ciprofloxacin (CIP)

• gentamicin (GEN)

• kanamycin (KAN)

• nalidixic acid (NAL)

• streptomycin (STR)

• tetracycline (TET)

• sulfisoxazole (SSS)

• trimethoprim/sulfamethoxazole (SXT)

The minimum inhibitory concentrations (MIC) and breakpoints were determined in accordance with guidelines established by the Clinical and Laboratory Standards Institute (CLSI) [16,17].

## Data analysis

GraphPad Prism 5 software (GraphPad Software, Inc. La Jolla, CA) was used for statistical analysis.

Between 2002-2007, 578 *Shigella *isolates were received and confirmed by ProvLab. The overall distribution of species included: *S*. *sonnei *54.7% (n = 316); *S. flexneri *33.9% (n = 196); *S*. *boydii *7.6% (n = 44); *S*. *dysenteriae *3.8% (n = 22). Twenty nine *S. flexneri *and 79 *S. sonnei *were not archived (stored and cataloged); three *S. flexneri *could not be cultured; 15 *S. sonnei *belonged to four outbreaks and were removed as they had the same antibiogram as the index isolate for each outbreak (nine *S. sonnei *isolates in 2006 and six *S. sonnei *isolates in 2007). All but four *S. flexneri *and *S. sonnei *isolates were isolates from stool specimens; two *S*. *sonnei *isolates from blood, and two *S. flexneri *isolates were from blood and urine. Of the 386 *S. flexneri *and *S. sonnei *isolates, 74.9% (n = 289) were associated with international travel; 12.7% (n = 49) associated with domestic exposure within North America; 12.4% (n = 48) unknown travel history or origin of acquisition.

Rate calculations from Alberta population data were utilized to ensure no bias to study. The data set lacks a true denominator for all specimens received and tested. *S. flexneri *rates ranged from 0.70 to 1.21 per 100,000, and *S. sonnei *rates ranged from 1.10 to 1.98 per 100,000 per annum. The majority of travel cases for *S*. *flexneri *were from Central America (32.3% [53/164]), the Indian subcontinent (22.6% [37/164]) and North America (8.5% [14/164]). The majority of *S*. *sonnei *cases were from Central America (39.2% [87/222]), North America (15.8% [35/222]), and the Indian subcontinent (11.3% [25/222]).

Of the 196 *S. flexneri *isolates, as described above 164 were available for analysis, while 29 were not archived and 3 did not grow. The most common ST for *S. flexneri *was ST2 (37.8% [62/164]) with 40.3% (25/62) of the ST2 isolates originating from Central America. Of the *S*. *flexneri *isolates from the Indian subcontinent the two most common STs were ST2 (40.5% [15/37]) and ST6 (35.1% [13/37]). The most common phage type for *S. sonnei *was S1 (65.8% [146/222]) with (38.4% [56/146]of S1 isolates from Central America.

Only 1.2% (n = 2) *S. flexneri *and 8.1% (n = 18) *S. sonnei *isolates were pan-susceptible to all antibiotics tested. All *S. flexneri *isolates were susceptible to AMI, GEN, AMC, KAN, FOX, TIO, AXO. All the *S. sonnei *were resistant to AMP, CHL, NAL, STR, TET and SXT (Table 1).

**Table 1:** Travel history and frequency of antimicrobial resistance of *Shigella *isolates in Alberta, 2002-2007^A^

	North America	Central America	South America	Africa	Middle East	Indian subcontinent	Far East Asia	Unknown	Western Hemisphere	Eastern Hemisphere
***Shigella flexneri***	**N = 14**	**N = 53**	**N = 6**	**N = 27**	**N = 2**	**N = 37**	**N = 8**	**N = 17**	**N = 73**	**N = 74**

	n(%)	n(%)	n(%)	n(%)	n(%)	n(%)	n(%)	n(%)	n(%)	n(%)

Streptomycin	7(50)	30(57)	3(50)	12(44)	2(100)	32(86)	6(75)	14(82)	40(55)	52(70)

Ampicillin	7(50)	39(74)	4(67)	22(81)	2(100)	23(62)	7(88)	14(82)	50(68)	54(73)

Trimethoprim-sulfamethoxazole	7(50)	21(40)	3(50)	17(63)	2(100)	26(70)	6(75)	14(82)	31(42)	51(69)

Sulfisoxazole	7(50)	25(47)	3(50)	21(78)	2(100)	26(70)	8(100)	14(82)	35(48)	57(77)

Chloramphenicol	8(57)	35(66)	4(67)	22(81)	2(100)	24(65)	6(75)	12(71)	47(64)	54(73)

Ciprofloxacin	1(7)	0(0)	0(0)	0(0)	0(0)	7(19)^1^	0(0)	0(0)	1(1)	7(9)

Nalidixic acid	1(7)	0(0)	0(0)	0(0)	0(0)	21(57)	0(0)	3(18)	1(1)	21(28)

Tetracycline	13(93)	51(96)	6(100)	25(93)	2(100)	37(100)	6(75)	17(100)	70(96)	70(95)

***Shigella sonnei***	**N = 35**	**N = 87**	**N = 14**	**N = 16**	**N = 2**	**N = 25**	**N = 12**	**N = 31**	**N = 136**	**N = 55**

Gentamicin	0(0)	0(0)	1(7)	0(0)	0(0)	0(0)	0(0)	1(3)	1(1)	0(0)

Streptomycin	34(97)	71(82)	7(50)	16(100)	2(100)	24(96)	10(83)	25(81)	109(80)	52(95)

Ampicillin	8(23)	30(34)	9(64)	1(6)	0(0)	1(4)	2(17)	19(61)	47(35)	4(7)

Amoxicillin/clavulanic acid	0(0)	0(0)	0(0)	0(0)	0(0)	0(0)	0(0)	1(3)	0(0)	0(0)

Ceftiofur	0(0)	0(0)	0(0)	0(0)	0(0)	0(0)	0(0)	2(6)	0(0)	0(0)

Ceftriaxone	0(0)	0(0)	0(0)	0(0)	0(0)	0(0)	0(0)	2(6)	0(0)	0(0)

Trimethoprim-sulfamethoxazole	26(74)	62(71)	14(100)	16(100)	2(100)	24(96)	11(82)	19(61)	99(73)	53(96)

Sulfisoxazole	30(86)	64(74)	14(100)	15(94)	2(100)	25(100)	10(83)	24(77)	105(77)	52(95)

Chloramphenicol	1(3)	0(0)	8(57)	0(0)	0(0)	1(4)	1(8)	0(0)	9(7)	2(4)

Ciprofloxacin	0(0)	0(0)	0(0)	0(0)	0(0)	0(0)	0(0)	0(0)	0(0)	0(0)

Nalidixic acid	4(11)	4(5)	0(0)	0(0)	0(0)	20(80)	0(0)	5(16)	8(6)	20(36)

Tetracycline	23(66)	57(66)	7(50)	15(94)	2(100)	25(100)	9(75)	14(45)	91(67)	51(93)

^A^Data for antimicrobial susceptible isolates are not shown

When median MICs were analyzed for all agents the following changes were identified as in Table 2. For *S. flexneri *median MICs were within two dilutions for most agents over the study period. Exceptions were for the following agents; AMP (increase), CHL (increase), SXT (increase and following drop), and SSS (decrease). For *S. sonnei*, median MICs were within two dilutions for most agents over the study period with the following exceptions; exception of AMP (decrease).

**Table 2:** Median MICs of antimicrobial agents in *S*. *flexneri *and *S*. *sonnei *per year.

		AMI	AMP	AMC	AXO	CHL	CIP	SXT	FOX	GEN	KAN	NAL	SSS	STR	TET	TIO
***S. flexneri***	**Total per year (n)**															

2002	10	4	2	2	< = 0.25	0.5	< = 0.015	< = 0.12	2	1	< = 8	1	> 256	< = 32	> 32	< = 0.12

2003	28	2	> 32	8	< = 0.25	> 32	< = 0.015	> 4	2	0.5	< = 8	1	> 256	> 64	> 32	0.25

2004	38	2	> 32	8	< = 0.25	32	< = 0.015	> 4	2	0.5	< = 8	1	> 256	> 64	> 32	< = 0.12

2005	35	2	> 32	8	< = 0.25	> 32	< = 0.015	> 4	4	0.5	< = 8	1	> 256	64	> 32	0.25

2006	22	2	> 32	8	< = 0.25	32	< = 0.015	0.25	2	0.5	< = 8	2	> 256	64	> 32	< = 0.12

2007	31	2	> 32	8	< = 0.25	32	< = 0.015	0.25	4	0.5	< = 8	2	< = 16	64	> 32	< = 0.12

																

***S. sonnei***	**Total per year (n)**															

2002	12	2	32	4	< = 0.25	4	< = 0.015	> 4	1	1	< = 8	1	> 256	> 64	> 32	0.25

2003	27	2	32	4	< = 0.25	4	< = 0.015	> 4	1	1	< = 8	1	> 256	> 64	> 32	0.25

2004	35	2	2	4	< = 0.25	4	< = 0.015	> 4	1	0.5	< = 8	1	> 256	> 64	> 32	0.25

2005	60	2	2	4	< = 0.25	8	< = 0.015	> 4	2	1	< = 8	1	> 256	> 64	> 32	0.25

2006	26	2	2	2	< = 0.25	4	< = 0.015	> 4	2	0.5	< = 8	1	> 256	> 64	> 32	0.25

2007	19	2	2	2	< = 0.25	4	< = 0.015	> 4	2	0.5	< = 8	2	> 256	> 64	> 32	0.25

amikacin (AMI), amoxicillin/clavulanic acid (AMC), ampicillin (AMP), cefoxitin (FOX), ceftiofur (TIO), ceftriaxone (AXO), chloramphenicol (CHL), ciprofloxacin (CIP), gentamicin (GEN), kanamycin (KAN), nalidixic acid (NAL), streptomycin (STR), tetracycline (TET), sulfisoxazole (SSS), trimethoprim/sulfamethoxazole (SXT)

**Figure 1:**
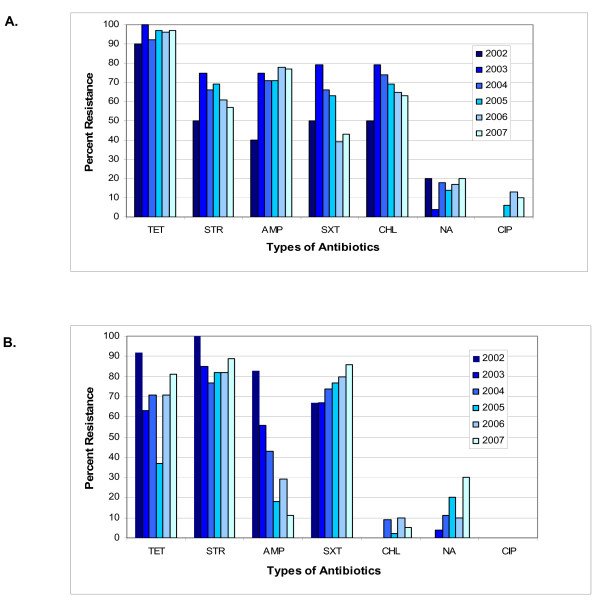
**Frequency of antimicrobial resistance of study isolates from Alberta 2002-2007: 1 a) *S. flexneri *(n = 164); and 1b) *S. sonnei *(n = 222)**.

When data was combined for all years, the NAL and CIP resistance was 20.1% (33/164) and 14.9% (33/222) for *S. flexneri *and *S. sonnei *respectively. CIP resistance was identified only in *S. flexneri *isolates (4.9%, 8/164) when averaged over the six-year study period (Fisher's exact test, p = 0.001) (Figure 1a and 1b) CIP resistance in *S. flexneri *was not steady but instead was most evident in the years 2005, 2006, and 2007 (Figure 1a). Combined CIP and NAL resistance was related to travel to the Indian subcontinent for *S*. *flexneri *(84.8%, 28/37) and *S*. *sonnei *(80.0%, 20/25) (Fisher's exact test, p < 0.0001). The proportion of antibiotic resistance was constant over six years except for *S. sonnei*, where AMP resistance decreased from 83% in 2002 to 11% in 2007 (p < 0.0001, χ^2 ^= 36.52, df = 5) and NAL resistance increased from 0% in 2002 to 30% in 2007 (p = 0.0168, χ^2 ^= 13.82, df = 5).

At the study onset, treatment guidelines suggested a fluoroquinolone for acute traveler's diarrhea regardless of travel location. It is possible that some CIP resistance was underestimated in 2002-2003 due to the smaller number of isolates tested. By 2009, treatment guidelines for acute traveler's diarrhea (outside of Latin America and Africa) suggested azithromycin or a fluoroquinolone [18,19]. Data also suggests that azithromycin resistance may be emerging and resistance rates of 16% have been recently described in Bangladesh [20]. These studies indicate that travel to the Indian subcontinent, in patients returning to Western Canada with traveler's diarrhea should be determined to guide initial empiric treatment options; especially for severe infections because the association of *S*. *flexneri *and *S*. *sonnei *isolates from this region with fluoroquinolone and potential macrolide resistance [13,21]. Although CIP resistance was described only in *S*. *flexneri*, we should remain vigilant for developing *gyrA *and *parC *mutations as well as the presence of plasmid mediated quinolone resistance determinants (PMQR) genes that may lead to increasing rates of CIP resistance in travel-related *Shigella *isolates which are beginning to emerge globally [4,22].

There are multiple factors that may have lead to CIP and NAL resistance in *Shigella *species originating from the Indian subcontinent [21]. It is possible that part of this emerging resistance may be associated with the increasing dominance of specific STs or clones of *Shigella*. Both this study and other work have identified a dominance of *S*. *flexneri *STs 2 and 6 in isolates of Indian origin and cases of traveler's diarrhea associated with the Indian subcontinent [23]. One factor driving multi-drug resistance in the Indian subcontinent may be the emergence of specific clones within these dominant STs [24]. Therefore, the identification of clonal groups within Alberta strains may be a powerful tool for tracking the development of drug-resistance in *Shigella *isolates from future cases of traveler's diarrhea.

## Competing interests

The authors declare that they have no competing interests.

## Authors' contributions

SJD, CL, MA, CF, and ML participated in data analysis and interpretation of susceptibility and travel data, drafted and revised paper, and made follow-up revisions to submission. CL, CF performed susceptibility testing on isolates, and interpreted/analyzed this data. CL collated, analyzed, and interpreted travel history data. LS, BF participated in susceptibility testing on isolates, and reviewed paper. KS, DE collaborated for travel history data, and reviewed/edited paper. ML, CL conceived study design. All authors read and approved the final manuscript draft.
